# Impeding ^99^Tc(IV) mobility in novel waste forms

**DOI:** 10.1038/ncomms12067

**Published:** 2016-06-30

**Authors:** Mal-Soon Lee, Wooyong Um, Guohui Wang, Albert A. Kruger, Wayne W. Lukens, Roger Rousseau, Vassiliki-Alexandra Glezakou

**Affiliations:** 1 Fundamental and Computational Sciences Directorate, Pacific Northwest National Laboratory, Richland, Washington 99352, USA; 2 Energy and Environment Directorate, Pacific Northwest National Laboratory, Richland, Washington 99352, USA; 3 Pohang University of Science and Technology, Pohang 37673, South Korea; 4 United States Department of Energy, Office of River Protection, Richland, Washington 99352, USA; 5 Lawrence Berkeley National Laboratory, Berkeley, California 94720, USA

## Abstract

Technetium (^99^Tc) is an abundant, long-lived radioactive fission product whose mobility in the subsurface is largely governed by its oxidation state. Tc immobilization is crucial for radioactive waste management and environmental remediation. Tc(IV) incorporation in spinels has been proposed as a novel method to increase Tc retention in glass waste forms during vitrification. However, experiments under high-temperature and oxic conditions show reoxidation of Tc(IV) to volatile pertechnetate, Tc(VII). Here we examine this problem with *ab initio* molecular dynamics simulations and propose that, at elevated temperatures, doping with first row transition metal can significantly enhance Tc retention in magnetite in the order Co>Zn>Ni. Experiments with doped spinels at 700 °C provide quantitative confirmation of the theoretical predictions in the same order. This work highlights the power of modern, state-of-the-art simulations to provide essential insights and generate theory-inspired design criteria of complex materials at elevated temperatures.

## Abstract (web summary)

Technetium-99 retention in spinel-containing glass is a promising strategy for radioactive waste management, but volatility is still an issue. Here, the authors show that doping magnetite with 1st row transition metals enhances technetium retention by altering the redox capacity of the Tc-containing spinel.

## Graphical abstract

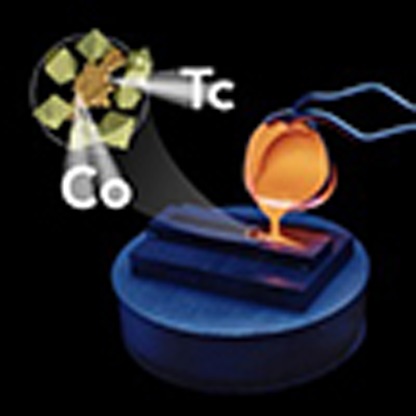

Technetium (^99^Tc) is an abundant long-lived radioactive fission product present in used nuclear fuel and waste generated from nuclear fuel reprocessing. Owing to its long half-life (2.1 × 10^5^ years) and relatively high fission yield (*∼*6*%*), ^99^Tc can generate the greatest radiation dose in the vicinity of a waste repository, and for a much longer time compared with other fission products, such as ^90^Sr and ^137^Cs (with half-life ∼30 years)^1^,^2^. In addition, TcO_4_^−^ is highly soluble and weakly adsorbed in the near-field, while Tc(IV) is highly adsorbable to geological materials and clays^3^. Thus, migration of Tc from a waste repository may be prevented by immobilizing Tc(IV) in durable waste forms, such as glass or ceramic materials^4^,^5^,^6^. Although Tc(VII)O_4_^−^ is the most stable Tc species under aerobic conditions, it is highly volatile at glass vitrification temperatures (∼1,200 °C), leading to poor Tc retention in the final waste glass^4^,^5^,^6^,^7^. Retention of Tc in the glass is generally improved by reducing conditions since Tc(IV) is less volatile^6^,^8^,^9^. Tc(VII) may be effectively reduced to Tc(IV) by Fe(II) in oxide and sulfide minerals or by Fe(II) adsorbed to mineral surfaces such as iron oxides or aluminium oxides^9^,^10^,^11^,^12^,^13^,^14^,^15^,^16^,^17^. However, retention of Tc is still limited because of re-oxidation of Tc(IV) back to Tc(VII) (refs 18, 19, 20, 21). Consequently, simply reducing Tc(VII) to Tc(IV) before vitrification is unlikely to stabilize Tc and prevent its volatilization as Tc(VII). An alternative approach would be to trap Tc(IV) in the lattice of a metal oxide by co-precipitation. Spinels are attractive targets for Tc stabilization during vitrification because of their physical and chemical stability under the high temperatures used in preparing borosilicate glasses^4^,^6^. In this respect, efficient incorporation and high retention of Tc by glass-incorporated spinels is very important for radioactive waste management and offers substantial economic benefit because of reduction in the amount of glass needed to immobilize ^99^Tc.

Magnetite (Fe_3_O_4_) has a cubic inverse spinel structure, where the oxygen anions form a slightly distorted face-centred-cubic sublattice and the iron cations occupy tetrahedral and octahedral interstitial sites. In the [001] direction, two types of layer stacking occur: *A* layers with tetrahedral Fe(III) and *B* layers with O and octahedral Fe(II)/Fe(III) (see Fig. 1). Marshall *et al*.^8^ showed that Tc(VII) can be reduced to Tc(IV) and incorporated into the magnetite structure under high pH conditions (pH 10.5−13.1). They also observed that Tc(IV) incorporation occurred at the octahedral sites and remobilization of Tc(IV) was limited during subsequent air oxidation. Kobayashi *et al*. observed Tc(IV) incorporation into the magnetite structure at pH 6 and pH 7.5 (ref. 22). However, magnetite oxidizes to maghemite (*γ*−Fe_2_O_3_) in oxic conditions or under high temperature through maghemitization, where all the Fe(II) atoms oxidize to Fe(III), while the oxygen sublattice remains unchanged^23^. When maghematization takes place, iron atoms diffuse towards the surface, leaving octahedral cation vacancies^23^,^24^. As a result, maghematization could lead to re-oxidation of Tc(IV) because of the increase in Fe(III), a highly efficient oxidizing agent^8^. Sidhu *et al*.^23^ suggested that incorporation of trace elements into magnetite stabilizes Fe(II) and suppresses maghematization by decreasing electron mobility. The majority of experimental studies on Tc retention are conducted at low temperatures, while theoretical studies employ static structural models that neglect temperature effects. Under these conditions, these studies cannot address Tc volatilization during vitrification that leads to poor Tc retention in the glass waste form. Thus, elucidation of high-temperature effects is important for understanding Tc retention by magnetite at elevated temperatures. *Ab initio* molecular dynamics (AIMD) simulations can describe the temperature effects on the change in structure, bonding and associated change in the oxidation states of Tc and Fe, which ultimately affects Tc retention. Here our simulations indeed show that leaching of Tc is accompanied with re-oxidation of Tc(IV) to Tc(VII) at high temperatures, but it can be suppressed by doping. We propose that inclusion of first transition metal dopants (Co, Zn and Ni) significantly improves Tc retention in magnetite at high temperature. Quantitative confirmation is further provided by X-ray absorption near edge structure (XANES) measurements and gravimetric analysis.

## Results

### Temperature effects on Tc(IV)-incorporated spinel

**Figure 1:**
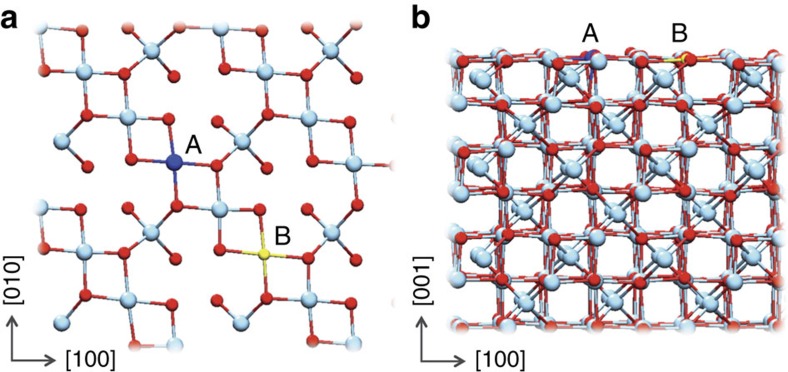
The B-truncated (octahedral Fe) Fe_3_O_4_(001) structure. (**a**) Top view and (**b**) side view of surface structure. Red and cyan circles represent oxygen and iron, respectively. A (blue circle) can be either Fe or Tc and B (yellow circle) can be either Fe or an impurity atom (Ni/Zn/Co).

The Fe_3_O_4_(001) surface has been studied extensively^25^,^26^,^27^,^28^,^29^,^30^. Pentcheva *et al*.^28^ compiled a phase diagram for the Fe_3_O_4_(001) surface in an *ab initio* thermodynamics study showing that the most stable surface structure is a *B*-terminated surface with octahedral iron and oxygen atoms forming a wave-like structure along the (001) direction. On the basis of these results, we generated a Fe_3_O_4_(001) model of a *B*-terminated surface (Fig. 1) that we let fully relax. Below the Verwey transition (125 K), the surface possesses a permanent dipole that has been shown to drive the formation of surface defects in these types of material^25^. However, above this temperature, magnetite is metallic, and the surface dipole is quenched^31^ and is not likely to affect surface charge defects. Details on the computational models and methods can be found in the Methods section.

**Figure 2:**
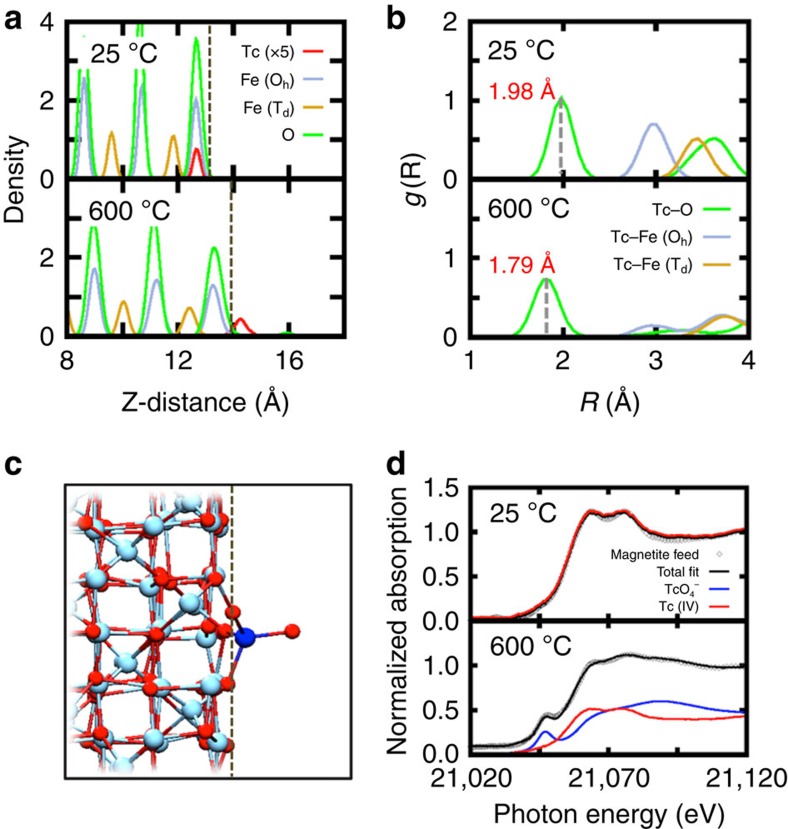
Structural properties and XANES spectra in the presence of Tc. (**a**) Atomic-density profile showing atomic arrangement along the *z*-direction at 25 and 600 °C obtained from AIMD simulations, where dotted vertical line denotes the magnetite surface. (**b**) Pair distribution function g(R) obtained from AIMD simulation trajectories at 25 and 600 °C. (**c**) Snapshot of the structure at 600 °C from AIMD trajectories where a blue circle represents Tc, red for O and cyan for Fe. The dotted vertical line denotes the magnetite surface. (**d**) Normalized XANES spectra at 25 and 600 °C.

To assess the temperature effects on Tc retention in magnetite, we replaced one octahedral Fe on the surface with Tc and performed AIMD simulations at two different temperatures, 25 and 600 °C, representing the ambient and the lower-end temperature range of the vitrification process, respectively. Figure 2a shows the calculated atomic density profiles of the different species with Tc scaled by 5 for clarity. The dotted grey line denotes the edge of the magnetite surface defined by the average position of the topmost oxygen atoms. At 25 °C, Tc stays within the top surface layer for the duration of the simulation (∼20 ps). Computation of pair distribution functions, *g*(R), reveals that, on average, surface-incorporated Tc has five nearest O neighbours with an average Tc–O distance 1.98 Å (Fig. 2b upper panel, Supplementary Fig. 1 and Supplementary Table 1). Additional exploratory simulations with Tc in an inner lattice position show a *g*(R) maximum at 2.01 Å for the Tc–O distance, compatible with the reduced Tc(IV) in magnetite (Supplementary Table 2). According to X-ray absorption fine structure (XAFS) analysis, the Tc(IV)–O distance is ∼2.0 Å (see Supplementary Table 2 and (refs 22, 32) for additional structural parameters). These observations imply that (i) within the surface layer the oxidation state of Tc is essentially Tc(IV) and (ii) at 25 °C (the glass-feed stage) reduced Tc(IV) is the prevalent oxidation state. However, the completely opposite picture emerges at high temperatures, 600 °C or higher. Tc moves above the surface, dragging coordinating surface oxygens along with it (see Supplementary Movie 1). The local Tc geometry is consistent with a tetrahedral Tc(VII)O_4_^−^ species, with two or three of the coordinating oxygens dynamically connected to Fe atoms on the surface (Fig. 2c). Analysis of *g*(R) for Tc–O pairs shows a peak at 1.79 Å (Fig. 2b, lower panel), an almost 10% reduction compared with the Tc(IV)–O distance at 25 °C. This change is compatible with the shorter Tc(VII)−O distances of ∼1.75 Å as determined by XAFS (Supplementary Table 2 and refs 22, 33). From this observation, we infer, that beginning at 600 °C, Tc oxidation is in process, commensurate with the tetrahedrally coordinated Tc transitioning to TcO_4_^−^. In addition to the system with Tc at the surface, we also examined a system with Tc at an inner lattice site at 600 °C. The calculated *g*(R) gives the distance between Tc and O as 2.01 Å, consistent with the reduced Tc(IV) in magnetite. As shown in Supplementary Fig. 2, Tc remains in the same layer throughout the simulation timescale. When comparing the energetics of the configurations with Tc below or at the surface layer, the energy with Tc below the surface is 2.5 eV higher than when Tc is at the surface. This implies that there is a thermodynamic driving force that will eventually move Tc out to the surface.

Experimentally, Tc-magnetite samples, heated from room temperature to 600 °C and then cooled back to room temperature, were analysed to determine the Tc oxidation state using XANES as shown in Fig. 2d. In the figure, the grey diamonds and black line indicate the measured data and a linear combination fit, respectively, for Tc-magnetite samples, while the red and blue lines represent the contribution from Tc(IV) and Tc(VII), respectively. At 25 °C, the spectrum of the sample shows only Tc(IV) (feed, red) but no Tc(VII) (blue), indicating that all Tc in the sample is in its reduced form. In the sample heated to 600 °C, however, the spectrum shows a mixture of both Tc(IV) and Tc(VII). All these observations are compatible with the simulations.

### Effects of dopants on Tc retention

**Figure 3:**
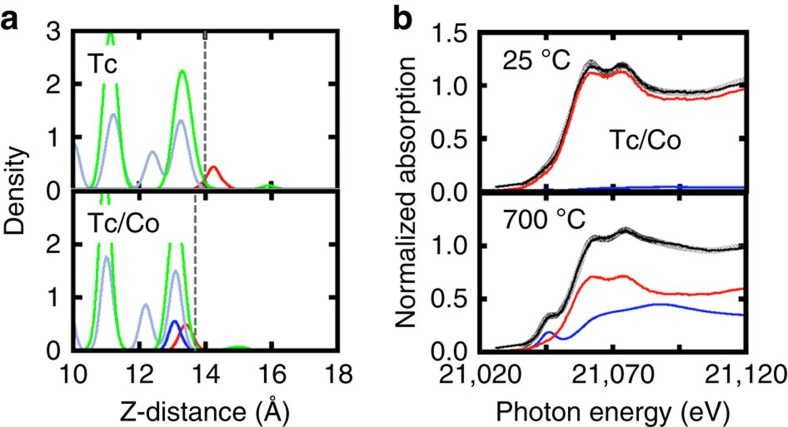
Atomic density and normalized XANES spectra with Co dopant. (**a**) Atomic density profiles with and without Co dopant at 600 °C. Red lines represent Tc, blue line for doped atom, grey lines for Fe and green lines for O. (**b**) Normalized XANES spectra for the Co-doped magnetite at 25 °C as made and treated at 700 °C. Colour codes used are the same as those shown in Fig. 2.

To simulate the effect of dopants on the Tc redox chemistry and immobilization, we modified the magnetite by substituting one surface Fe atom with Ni, Zn or Co (∼1% wt each) at a site close to Tc. This choice was motivated by earlier experiments by Sidhu *et al*.^23^, who observed stabilization of Fe(II) and suppression of maghematization when first row transition metal dopants were present in magnetite even at concentrations ∼1 wt %. The atomic density profiles along the surface normal from AIMD at 600 °C in the presence of the doping elements are shown in Fig. 3 (Co) and Supplementary Fig. 3 (Ni and Zn), exhibiting an increase in Tc retention in the order Co>Zn>Ni.

In the case of Ni, the Tc population is bi-modal where Tc remains mostly on top of the surface with only a small population within the top surface layer. In the case of Zn, the bi-modal Tc distribution is shifted towards a larger Tc population within the surface. Analysis of trajectories also shows that the distance between Tc and the coordinating O fluctuates between 1.71 and 1.92 Å, compatible with an equilibrium between Tc(VII) and Tc(IV) oxidation states (see Supplementary Fig. 4). This behaviour implies that Ni and Zn only partially, and to a similar degree, hinder Tc oxidation. Finally, in the presence of Co, Tc remains almost in its entirety within the surface at all times indicative of a Tc(IV) state. We examined Tc(IV) stabilization in the presence of Co by conducting a simulation starting with TcO_4_^−^ on top of the surface. As shown in Supplementary Movie 2, Tc(VII) rapidly migrates into the surface becoming Tc(IV), within 1.5 ps of simulation time.

To validate these findings, we prepared three different magnetite samples doped with ∼10% wt of Ni, Zn and Co. Details on the preparation of samples can be found in the Methods section. The samples were heated at 700 °C in a furnace for 1 h, and the remaining Tc was measured (see Supplementary Table 3). Gravimetric measurement showed that doping with Co resulted in the highest Tc retention (29% wt) compared with less than half that amount for Zn (12% wt) and ∼1/8 of that for Ni (4% wt). No detectable amount of Tc was found in the Tc-magnetite sample prepared without dopant and treated at 700 °C. We also performed XANES measurements for the samples prepared at 25 and 700 °C (see Supplementary Methods for details) and confirmed our theoretical prediction of the highest Tc retention with Co dopant at high temperature, as shown in Supplementary Table 4 and Fig. 3b and Supplementary Fig. 5.

### Equilibrium constants and free energy estimates

To best connect with the experimental observations, we determined the ratio of the equilibrium populations between the two different oxidation states of Tc(IV) ([Tc_in_]) and Tc(VII) ([Tc_out_]). This can be achieved by integrating the area under the atomic density profiles for Tc in Fig. 3. An equilibrium constant between the two populations, determined as the ratio *K*_eq_=[Tc_in_]/[Tc_out_], was used to calculate the Gibbs free energy for this equilibrium from the relation Δ*G=*−*RT* ln *K*_eq_, where *R* is the gas constant and *T* is the absolute temperature. Negative values indicate that the equilibrium favours a higher population of Tc(IV). Table 1 summarizes the computed values of *K*_eq_ and Δ*G*, as well as the measured Tc retention for the different doping agents Ni, Zn and Co. These results show a remarkable agreement between the theoretical prediction and experimental validations, not only in terms of relative order but also in magnitude. The underlying reason is based on the increase in the reducing capacity of the Tc-containing spinels upon doping. This can be quantified by the difference in energy between the Fermi level, *E*_F_, and the highest occupied molecular orbital (HOMO) of Tc *d* states, Δ*E*_gap_, see last column in Table 1. The calculated total and projected density of states (DOS) of the *d*-band for Tc and dopant are shown in Supplementary Fig. 6. Whereas in the case of Ni only a marginal stabilization of the Tc *d*-states occurs (small Δ*E*_gap_), in the case of Zn and Co, a much higher stabilization takes place that ultimately hinders Tc re-oxidation.

## Discussion

In conclusion, we propose that standard reduction potentials of transition metal ions relative to those of parent spinel, combined with their available oxidation states, can be a useful diagnostic tool for identifying appropriate additives. The reduction potential for magnetite ranges from +0.22 to +0.66 V (ref. 34), while those for Co^2+^, Ni^2+^ and Zn^2+^ are −0.28, −0.26 and −0.76 V, respectively^35^, and in principle Co^2+^ and Ni^2+^ should have similar and limited effect upon Tc retention, while Zn^2+^ should have a more pronounced influence. However, Co^2+^ with a wide range of redox values towards Co^3+^ 35,36(refs. 35, 36), it greatly increases the overall reducing capacity of the spinel material. This is reflected in the increased stabilization of the Tc *d*-states, see Table 1. Both simulations and experiment show that cobalt is by far the most effective additive for Tc retention compared with the undoped magnetite. We postulate that Tc retention, during the glass vitrification, can be controlled by balancing the redox capacity of oxide materials and doping agents. The current study underscores the impact of complex models incorporating both electronic structure and temperature effects that reveal the critical variables needed for predictive materials' design.

## Methods

### Density functional theory (DFT) parameters

Spin-polarized DFT simulations were performed with periodic boundary conditions (3D PBC) as implemented in the CP2K package^37^. The Perdew, Burke and Ernzerhof (PBE) generalized gradient approximation was used for the exchange-correlation functional^38^. The core electrons were described by the norm-conserving pseudopotentials^39^, while the valence wave functions were expanded in terms of double-zeta quality basis sets optimized for condensed systems to minimize linear dependencies and superposition errors^40^. An additional auxiliary plane wave basis set with a 500-Ry cutoff was used to calculate the electrostatic terms. The GGA+U scheme was used to provide more accurate electronic structure for the localized *d*-orbitals. The Hubbard parameter (U–J) of 3.5 eV was taken for the Fe 3*d* states, which results in a work function of 5.32 eV, in good agreement with that obtained by Pentcheva *et al*.^28^ Owing to large supercell simulations, the *Γ*-point approximation was used for the Brillouin zone integration.

### Computational models

To study Tc incorporation in magnetite with and without dopants, we used a 2 × 2 × 2 supercell in all simulations to minimize periodic images. Optimization of the bulk structure of magnetite had a cell parameter of 8.391 Å, which agrees well with experimental data (8.390 Å (ref. 41)). Using this optimized cell parameter, we constructed a magnetite(001) surface model terminated at an octahedral Fe sublattice, since it is known to be the most stable surface structure in magnetite. A more recent surface model was also considered^42^, but was found not to have significant impact on the present problem, see SI. Our model system consisted of a symmetric slab with seven octahedral and six tetrahedral Fe sublattices (384 atoms) with a vacuum region of 12.5 Å between slabs. To study Tc incorporation, one surface octahedral Fe was replaced with Tc, followed by structural optimization. We also optimized a structure with one octahedral Fe in the third layer replaced by Tc. For the doping studies, we substituted a surface Fe atom with Co, Ni or Zn (∼1 wt%) at a lattice position close to Tc. In all simulations, we fixed the atomic positions of the four bottom atomic layers.

### AIMD simulations

AIMD simulations were performed with and without Tc at 25 °C and with the dopants Co/Ni/Zn at 600 °C, with the Nosé–Hoover thermostat for NVT ensemble and a time step of 1.0 fs. Each simulation was equilibrated for at least 20–28 ps, and the last 10–12 ps of the trajectories was used for the analysis. Owing to the big computational cost of high-temperature simulations, we chose lower range of vitrification temperatures (600 °C), while experiments were performed at somewhat higher temperatures (∼700 °C).

### Spinel synthesis and XAFS analysis

Ni-, Zn- or Co-doped Tc-incorporated magnetite was synthesized at high pH (>13). Three solutions of 0.05 M Ni, Zn and Co in distilled deionized water (DDI) were prepared using analytical-grade NiCl_2_, ZnCl_2_ and CoCl_2_. Technetium solution (0.001 M) was prepared by spiking 10,000 p.p.m. NaTcO_4_ stock solution into 1 M NaOH solution. Synthesized Fe(OH)_2_ dry powder (0.09 g) was mixed with 5 ml of NiCl_2_, ZnCl_2_ or CoCl_2_ solution in 20-ml poly vials and shaken on an orbital shaker (120 r.p.m.) for 24 h at room temperature (RT). After 24 h shaking, 15 ml of the Tc-spiked 1-M NaOH was added to each vial and heated in an oven at 75 °C for 72 h. After cooling to RT, the precipitates were separated using 0.45-μm filters and washed using ∼120 ml DDI water immediately after filtering. The collected solid precipitates were air-dried at RT overnight and stored in glass vials. Strong microwave-assisted digestion with a solution consisting of 16 M HNO_3_ (17%), 12 M HCl (7%), 32 M HF (3.3%), 0.5 g H_3_BO_3_ (1.5%) and DDI water (71.2%) on a volume basis was used to determine the total Tc concentration in the final solid samples. For the 600 °C XANES sample, ∼5 g of Tc-magnetite was mixed with other basic glass feeds in a Pt crucible and heated in a furnace to 1,000 °C at 5 °C increase per minute. After air quenching, the final glass was pulverized and used for XANES analysis. Additional Tc XAFS samples were also prepared for Ni-, Zn- or Co-doped Tc-incorporated magnetite at room temperature without basic glass feeds and treated at 700 °C inside an oven. The XAFS spectra were collected at room temperature on Beamline 4-1 at the Stanford Synchrotron Radiation Laboratory. A Si(220) double-flat crystal monochromator was used, and the energy was calibrated by using the first inflection point of the Tc K edge spectrum of the Tc(VII) standard (KTcO_4_) defined as 21.044 keV. The XAFS spectra of Tc standards and Tc-magnetite samples were collected in transmission and fluorescence mode, respectively, at RT using a 13-element germanium detector. Data reduction and analysis were performed using the software IFEFFIT and Athena/Artemis^43^ after detector dead-time correction. The XANES spectra for Tc samples were fit using a linear combination of the XANES spectra of KTcO_4_ as the Tc(VII) standard spectrum and TcO_2_·2H_2_O as the Tc(IV) standard spectrum, see Supplementary Fig. 7 and Supplementary Methods for more details.

### Data availability

The authors declare that the data supporting the findings of this study are available within the article and its supplementary information files.

## Additional information

**How to cite this article:** Lee, M.-S. *et al*. Impeding ^99^Tc(IV) mobility in novel waste forms. *Nat. Commun.* 7:12067 doi: 10.1038/ncomms12067 (2016).

## Supplementary Material

Supplementary Information Supplementary Figures 1-7, Supplementary Tables 1-4, Supplementary Methods and Supplementary References.

Supplementary Movie 1 AIMD simulations at 600 °C showing leaching of Tc out of the magnetite surface, forming tetrahedral (TcO4)^−^.

Supplementary Movie 2 The movie is generated from AIMD simulations at 600 °C showing (TcO4)^−^ returning into the magnetite surface, in the presence of Co dopant.

## Figures and Tables

**Table 1:** Equilibrium constants and free energy estimates considering doping effects.

**Doping**	***K***_**eq**_	Δ***G*** **(kJ mol**^**−1**^**)**	**Exp. retention (%wt)**	Δ***E***_**gap**_**(eV)**
Tc	0.15	14.2	–	0.15
Tc/Ni	0.56	4.3	4	0.35
Tc/Zn	2.79	−7.7	12	1.03
Tc/Co	21.80	−23.1	29	1.12

Tc implies system without dopant. Relative populations determined as a ratio from the computed *K*_eq_ values. Experimental values correspond to the amount of Tc remaining in the doped magnetite after exposure to 700 °C. Δ*E*_gap_ represents the energy difference between the Fermi level *E*_F_ and the Tc HOMO energy from the projected DOS.
