# Supplementary material for: Impeding 99Tc(IV) mobility in novel waste forms
Source: Nat Commun. 2016 Jun 30;7:12067. doi: 10.1038/ncomms12067 (PMC4931311; doi:10.1038/ncomms12067)
Supplement: Supplementary Information — Supplementary Figures 1-7, Supplementary Tables 1-4, Supplementary Methods and Supplementary References. [file ncomms12067-s1.pdf]

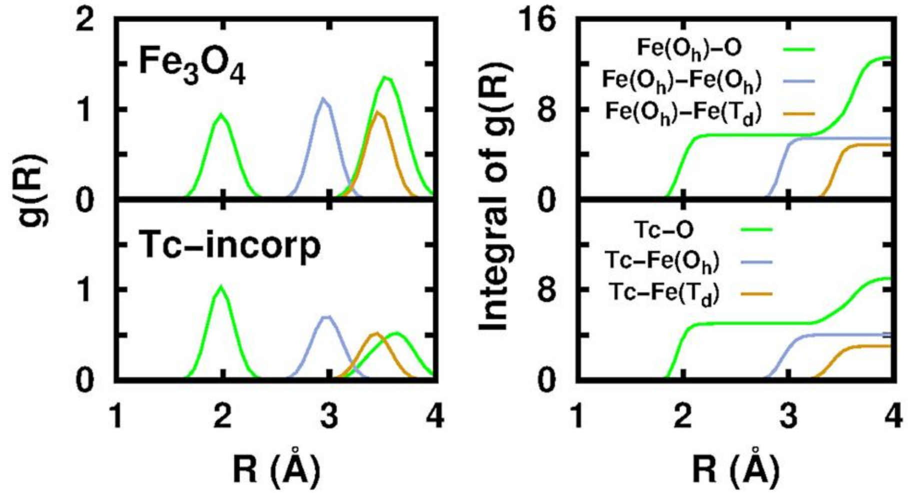

Supplementary Figure 1 | Structural properties of Tc-incorporated magnetite at 25 °C. Pair distribution function g(R) and its integral for magnetite without and with Tc at the surface obtained from AIMD simulation trajectories at 25 °C.

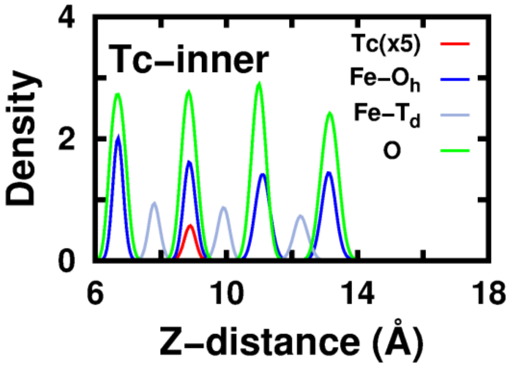

Supplementary Figure 2 | Tc-incorporation at an inner layer at 600 °C. Atomic density profile for Tc incorporated to the 3rd Fe(O_h) layer at 600 °C.

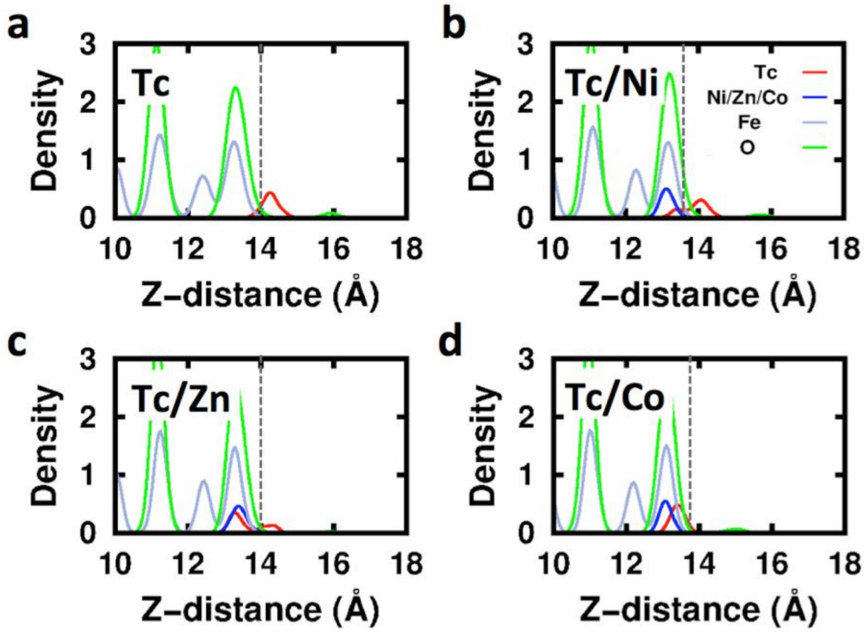

Supplementary Figure 3 | Atomic density profiles with different dopants at 600 °C. Atomic density profile along the z direction is shown **(a)** without dopant, **(b)** with Ni dopant, **(c)** with Zn dopant, and **(d)** with Co dopant in Tc-incorporated magnetite. Red lines represent Tc, blue lines the three different doping atoms (Ni, Zn, and Co), gray lines Fe atoms, and green lines O atoms. Vertical dotted lines represent the topmost edge of the magnetite surface.

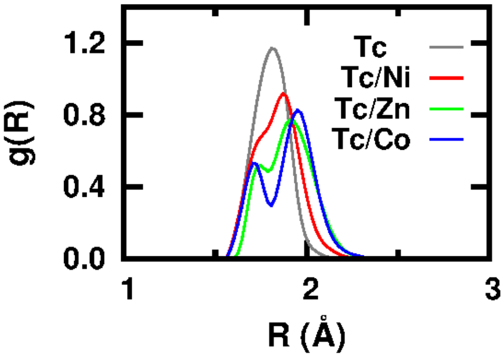

Supplementary Figure 4 | Pair distribution function in Tc-incorporated magnetite with and without dopant at 600 °C. Pair distribution function, g(R), between Tc and first O nearest neighbors in Tc-incorporated magnetite with and without doping elements, from AIMD simulation trajectories at 600 °C. The analysis shows a bi-modal distribution for systems with dopants and corroborates that Tc fluctuates between Tc(VI) and Tc(VII) states during simulation.

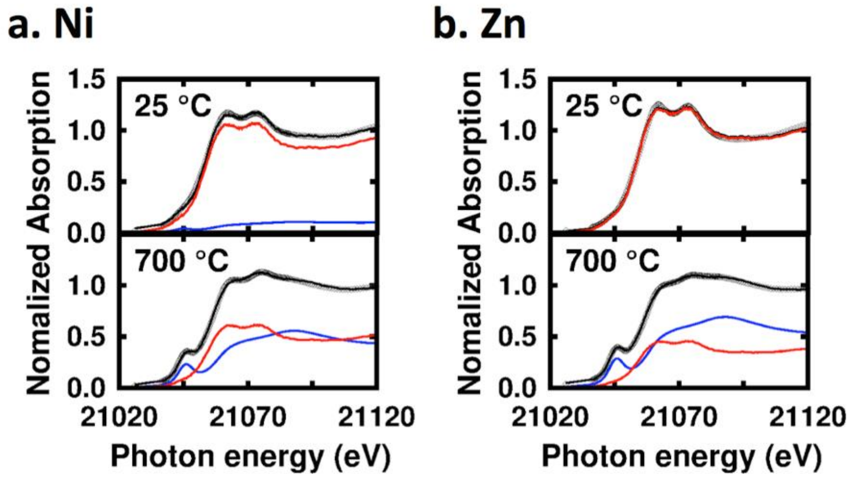

Supplementary Figure 5 | K-edge XANES spectra and fit at 25 and 600 °C for (a) Ni-doped and (b) Zn-doped Tc-magnetite. Color codes used are the same as those shown in Fig. 2.

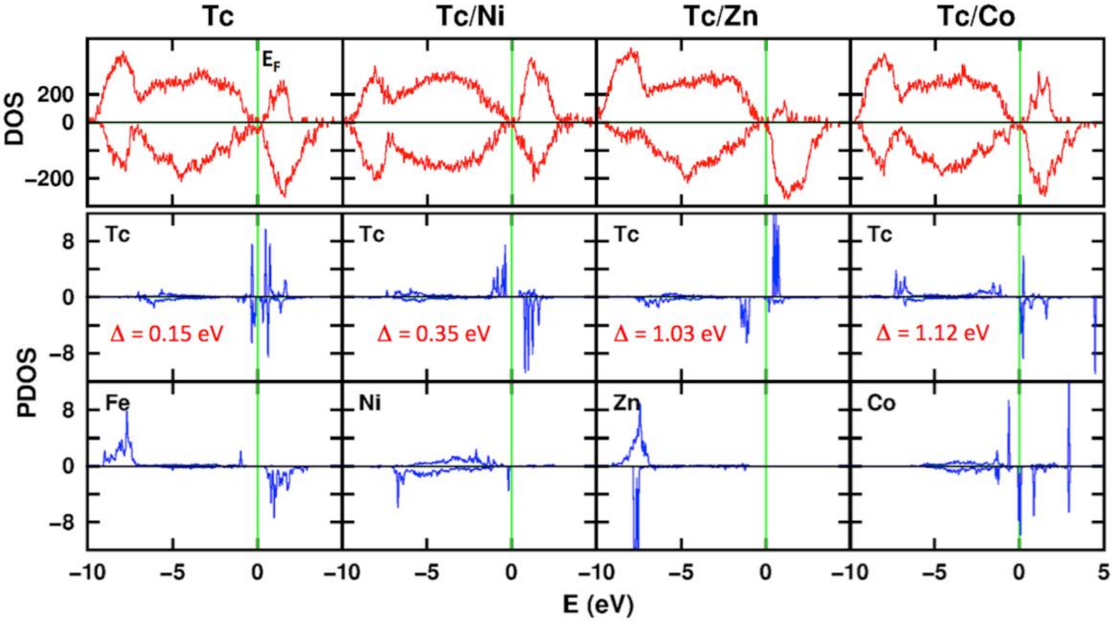

Supplementary Figure 6 | Calculated total density of states and projected density of states with and without dopant at 0 K. The top panel shows total density of states, middle panel for projected density of states of Tc *d*-band, and bottom panel for projected density of states of Fe/Ni/Zn/Co *d*-band. Green lines indicate Fermi energy. **Δ** gives the energy difference between Fermi level and the highest occupied molecular orbital of Tc *d*-band that allowed us to evaluate the overall redox capacity of the doped material.

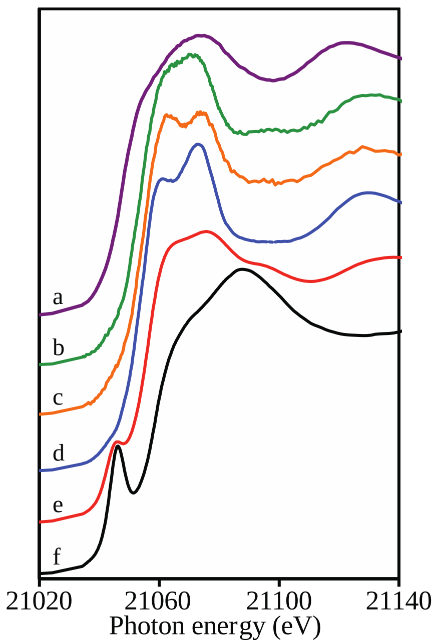

Supplementary Figure 7 | Tc K-edge XANES spectra of the reference compounds used for XANES fitting. From top to bottom, these are the Tc K-edge spectra of Tc_2S_7 (a), Tc(IV) EDTA (b), $TcO_2{\bullet}2H_2O$ (c), Tc(IV) gluconate (d), $PW_{11}Tc{=}O$ (e), TcO_4^- (f).

Supplementary Table 1. Comparison of coordination environment in the Tc-incorporated system obtained from radial distribution function calculations from AIMD simulations and experiments at 25 °C (See Supplementary Fig. 1).

Pair	Coordination #		Distance (Å)	
	Theory	Exp.	Theory	Exp.
**Tc-O**	5.0	5.2±0.2	1.98	2.00±0.01
**Tc-Fe(O_h)**	4.0	3.0±0.8	2.98	3.05±0.01
**Tc-Fe(T_d)**	3.0	3.0±0.8	3.45	3.48±0.01

Supplementary Table 2. Detailed EXAFS analysis for Tc coordination environment where EXAFS fitting parameters are given for Tc at 25 °C with [illegible]$_0^2$=0.9 (fixed) and ΔE=0. O_h denotes the octahedral and T_d the tetrahedral sites. Also see Refs. 1, 2.

Neighbor	Coord #	Distance (Å)	σ^2 (Å^2)	p	Fe_3O_4 Structure	Tc Structure
**O**	0.6±0.2	1.74±0.03	0.0040(6)	0.011	6 O @2.06 Å	TcO_4^-: 4 O @1.75 Å[a]
**O**	5.2±0.2	1.997±0.007	0.0040(6)	<0.001		Tc(IV):6 O @2.0 Å[b]
**Fe-O_h**	3.0±0.8	3.05±0.01	0.006(1)	0.001	6 Fe @2.97 Å	
**Tc-O_h**	0.8±0.5	3.05±0.01	0.006(1)	0.111		
**Fe-T_d**	3.0±0.8	3.48±0.01	0.006(1)	0.002	6 Fe @3.48 Å	

Supplementary Table 3. Gravimetric analysis of Tc retention after treatment at 700 °C for 1 hour with different dopants in Tc-incorporated magnetite.

Dopant	Tc Conentration (μg/g)		Retention (%)
	Initial	Final	
**Ni**	18,200±700	726±30	4.0±0.4
**Zn**	15,500±200	1,930±20	12±0.1
**Co**	14,600±400	4,280±100	29±0.3

Supplementary Table 4. Tc K-edge XANES results[a]

Dopant	T(°C)	TcO_4^-	*p*	Tc(IV)	*p*
Ni	25	0.07±0.03	0.007	0.93±0.02	<0.001
	700	0.49±0.02	<0.001	0.51±0.01	<0.001
Zn	25	0.00±0.03	1.000	1.00±0.02	<0.001
	700	0.61±0.01	<0.001	0.39±0.01	<0.001
Co	25	0.03±0.03	0.402	0.97±0.02	<0.001
	700	0.40±0.02	<0.001	0.60±0.01	<0.001

a) Standard deviation of the fit is given. The value of p is the probability that the improvement to the fit from including this spectrum is due to random error. Components with $p < 0.05$ are significant at the two-sigma level and those with $p < 0.01$ are significant at the 3 sigma level.

Supplementary Methods: K-edge XANES Experiments

The magnetite synthesis followed a standard method similar to the Schikorr reaction that describes the conversion of $Fe(OH)_2$ into Fe_3O_4. [3-5]
After $Fe(OH)_2$ solution was prepared at low pH (~2), the pH was adjusted to ~13 by adding Tc-spiked NaOH (0.1 M) solution, and the slurry was cooked at 75°C. Increasing pH close to 13 by direct addition of NaOH caused an immediate magnetite precipitate in which Tc was reduced and simultaneously incorporated into the fresh magnetite precipitate.

Data were obtained at SSRL BL 11-2 at the Tc K edge (21044 eV). XANES data were obtained from 200 eV below the edge to 1000 eV above the edge; the data from 75 eV below the edge to 200 eV above the edge was obtained with 0.5 eV spacing. The rest of the data points are widely spaced (50 eV) and were used for the pre- and post-edge correction. EXAFS data were obtained with the same pre-edge energy spacing, and the spacing above the edge was obtained with a k-spacing of 0.05, and the collection time was weighted by k^3. The monochromator was detuned 50% to reduce the harmonic content of the beam. Transmission data were obtained using Ar filled ion chambers. Fluorescence data was obtained using a 100 element Ge detector and were corrected for detector dead time. Data were reduced from raw data to spectra using SixPack. Spectra were normalized using Artemis. Sample spectra were convolved with a 1.2 eV Gaussian to match the resolution of the standard spectra. Normalized XANES spectra were fit using standard spectra in the locally written program “fites”. XANES spectra were carefully energy calibrated using TcO_4^- adsorbed on Reillex-HPQ as the energy reference. The sample XANES spectra were allowed to vary in energy during fitting. The XANES spectral resolution is 7 eV based on the width of the TcO_4^- pre-edge peak, so each spectrum possesses 20 independent data points (range of the spectrum/resolution).

The significance of each standard spectrum in the final fit was assessed using an F-test, which provides the probability, p, that the improvement in the fit due to adding a standard spectrum is due to random error (primarily noise in this case). The p-factor is mainly useful for excluding standards that do not significantly improve the fit. Samples with p greater than 0.05 do not significantly improve the fit and should not be considered to be observed (2 σ test). Standards with p between 0.05 and 0.01 significantly improve the fit, but the improvement is not very significant. Standards with p less than 0.01 significantly improve the fit, and standards with p less than 0.001 very significantly improve the fit.

Five standard spectra were used in the initial fitting of the sample XANES spectra: TcO_4^-, $TcO_2{\bullet}2H_2O$, Tc(V) polyoxometalate, Tc(IV) gluconate, and Tc(IV)

EDTA complex. The latter three standards represent Tc(IV) with varying degrees of disorder in the first shell of 6 oxygen atoms. In Tc(IV) gluconate, the first shell consist of 6 oxygen atoms 2.0 Å from the Tc center in an octahedral geometry. In $TcO_2 \bullet 2H_2O$, there are 4 oxygen atoms 2.0 Å from the Tc center and two oxygen atoms at a longer distance, ~2.4 Å. In Tc(IV) EDTA, the first shell is quite distorted with two oxygen atoms at 2 Å, two oxygen atoms at a somewhat longer distance, and two nitrogen atoms still further away. These changes in the local environment of Tc produce changes in the structure of the Tc K-edge XANES spectrum as illustrated in Supplementary Figure 7.

The Tc-XANES spectra were fit in the following way. Initially, all five standard spectra were used to fit each spectrum. Any non-pertechnetate standard spectrum for which the contribution was less than two standard deviations was removed, and the fitting process was repeated until TcO_4^- and a single Tc(IV) standard remained. In these samples, only $TcO_2 \bullet xH_2O$ contributed significantly. The purpose of removing the Tc(IV) spectra that are not significant is to determine accurate standard deviations for the amount of Tc(IV) in the sample. If more than one Tc(IV) standard is used, the standard deviation of each becomes very large since their contributions are strongly correlated. In other words, the spectra of all of the Tc(IV) species are similar, so it is difficult to precisely determine how much of each Tc(IV) standard is present in any sample. The fact that the Tc(IV) EDTA standard spectrum fit the data well does not necessarily mean that the samples contain Tc(IV) EDTA. Rather, the fact that the Tc(IV) EDTA reference spectrum fits well means that the Tc environment in the sample resembles the $Tc_2(\mu\text{-}O)_2$ dimeric structure of Tc(IV) EDTA.

Supplementary References

1. Allen, P. G. *et al.* Technetium speciation in cement waste forms determined by X-ray absorption fine structure spectroscopy. *Radiochim. Acta* **76,** 77-86 (1997).
2. Lukens, W. W., Bucher, J. J., Edelstein, N. M. & Shuh, D. K. Products of pertechnetate radiolysis in highly alkaline solution: structure of $TcO_2 \cdot xH_2O$. *Environ. Sci. Technol.* **36,** 1124-1129 (2002).
3. Ardizzone, S. & Formaro, L. Temperature induced phase transformation of metastable $Fe(OH)_3$ in the presence of ferrous ions. *Mater. Chem. Phys.* **8,** 125-133 (1983).
4. Schikorr, G. Über Eisen(II)-hydroxyd und ein ferromagnetisches Eisen(III)-hydroxyd. *Z. Anorg. Allg. Chem.* **212,** 33-39 (1933).
5. Um, W., Wang, G., Jung, H. B., & Peterson, R. A. Technetium removal using Tc-goethite coprecipitation. Pacific Northwest National laboratory, PNNL-22967 (2013).
